# Partner notification in the context of HIV: an interest-analysis

**DOI:** 10.1186/s12981-015-0057-8

**Published:** 2015-05-05

**Authors:** Amos K Laar, Debra A DeBruin, Susan Craddock

**Affiliations:** Department of Population, Family, & Reproductive Health, School of Public Health, University of Ghana, Accra, Ghana; Center for Bioethics, University of Minnesota, Minneapolis, USA; Department of Gender, Women, and Sexuality Studies, University of Minnesota, Minneapolis, USA

**Keywords:** HIV, partner notification, interest-analysis, principles-based analysis

## Abstract

Codes of confidentiality play an essential role in the intimate discourses in many learned professions. Codes with various prescriptions exist. The Hippocratic Oath for example, prescribes rewards to the secret keeper, for keeping secret what ought to be kept secret, and punishments for failing. In public health practice, partner notification, arguably is one endeavor that tests the durability of this secret keeping doctrine of the health professional. We present an interest-analysis of partner notification in the context of HIV service rendition. Using principles-based analysis, the interests of the individual, the state/public health, and the bioethicist’s are discussed. The public health interests in partner notification, which are usually backed by state statutes and evidence, are premised on the theory that partners are entitled to knowledge. This theory posits that knowledge empowers individuals to avoid continuing risks; knowledge of infection allows for early treatment; and that knowledgeable partners can adapt their behavior to prevent further transmission of infection to others. However, persons infected with HIV often have counter interests. For instance, an infected person may desire to maintain the privacy of their health status from unnecessary disclosure because of the negative impacts of disclosure, or because notification without a matching access to HIV prevention and treatment services is detrimental. The interest of the bioethicist in this matter is to facilitate a resolution of these conflicted interests. Our analysis concludes that governmental interests are not absolute in comparison with the interests of the individual. We reiterate that any effort to morally balance the benefits of partner notification with its burdens ought to first recognize the multivalent nature of the interests at play.

## Introduction

One of the things that learned professions such as law, the clergy, medicine, and even public health have in common is codes of confidentiality. These codes ensure the sanctity of the intimate conversations that characterize them. In public health practice for example, client-provider confidentiality, has been treasured for ages [1]. The earliest explicit codification of confidentiality in the context of medicine is attributed to Hippocrates of Cos (460 BC – 370 BC). Titled “Oath of Hippocrates” the code prescribes rewards to the secret keeper, for living up to his obligations and punishments for those who do not (see extract below):

*“Whatsoever in the course of practice I see or hear (or even outside my practice in social intercourse) that ought never to be published abroad, I will not divulge, but consider such things to be holy secrets. “Now if I keep this oath and break it not, may I enjoy honor, in my life and art, among all men for all time; but if I transgress and forswear myself, may the opposite befall me…”* [2].

Millennia upon millennia, medical oaths continue to agree fundamentally with the tenets of the Hippocratic ethics. This is not to say that professionals’ obligation to keep the secrets of their clients has been polemics-free. Alan Brandt in his “*No Magic Bullet, the social history of venereal diseases in the United States …”* animates the vehemence with which physicians within the United States (US), and those from across the Atlantic debated the issue of partner notification [3]. Brandt recounts the various medical, military, and public health responses that have arisen over the years – including the incarceration of prostitutes during World War I to the establishment of the extensively debated premarital blood tests. Of note, Brandt calls for the destabilization of the naturalness with which efforts that combat sexually transmissible infections have centered on punishment for sexual misconduct.

Introduced for syphilis and then extended to include gonorrhea in the 1930s and 1940s [3,4], partner notification is now considered useful for a wide range of sexually transmissible infections (STIs) [5]. Partner notification is currently defined as the process whereby the sexual partner(s) of a case (an index patient) is/(are) identified and informed of their exposure, then invited to testing, counseling and, where necessary, treatment [6,7]. As a term, “partner notification” has undergone some metamorphoses in the past decade and-a-half. Prior to 1998, guidelines of the US Centers for Disease Control and Prevention (CDC) contained “contact tracing” or “partner notification”. These terms were dropped in 1998 for “partner counseling and referral services” [8]. The current CDC guidelines use “partner services” [6]. Related terminologies in current use include “expedited partner therapy”, “privilege to warn” “duty to warn” and “duty to disclose”. While many academics consider these as equivalent activities, public health practitioners consider them to be vastly different in most respects except for the principal goal [9]. Each has unique logistic and substantive issues. For instance, partner notification as is currently defined, requires that only sexual partners be notified that they have been in contact with a sexually transmissible infection. It is a confidential process: details of the index cases are known only to the health professionals treating them and are not divulged either to sexual partners or to disease notification systems. Contact tracing entails all the health care’s actions taken to trace and actively and systematically contact all the partners/contacts indicated by the index person as having had relationships with him/her at risk. Bayer and Toomey discriminate between contact tracing and duty to warn [10]. They note that the moral “duty to warn”, arose out of the clinical setting in which the physician knew the identity of the person deemed to be at risk. This approach provided a warrant for disclosure to endangered persons without the consent of the patient and could involve the revelation of the identity of the “threatening” party (the index patient). Bayer and Toomey lament that confusion regarding the approaches has led many to mischaracterize processes that are fundamentally voluntary as mandatory, and processes that respect confidentiality as invasive of privacy.

We note, however, that the terminology of partner notification differs between countries; it is used interchangeably with contract tracing in such countries as the United Kingdom and Australia. The global guidelines from the World Health Organization (WHO) [11] use partner notification. The WHO and the Joint United Nations Programme on HIV/AIDS (UNAIDS) recommend that partner notification be done on a voluntary basis [12]. Where partner notification has to be done without consent, the UNAIDS provides clear guidelines [13] such as: (i) The HIV-positive person in question has been thoroughly counselled; (ii) Counseling of the HIV-positive person has failed to achieve appropriate behavioral changes; (iii) The HIV-positive person has refused to notify, or consent to the notification of his/her partner(s); (iv) A real risk of HIV transmission to the partner(s) exists; (v) The HIV-positive person is given reasonable advance notice; (vi) The identity of the HIV-positive person is concealed from the partner(s), if this is possible in practice; (vii) Follow-up is provided to ensure support to those involved, as necessary.

As illustrative examples, we summarize below the global outlook of partner notification using relevant resources from the US, Europe, as well as the WHO global guidelines. These guidelines provide background and basis for the roll out of partner notification services globally. First, a technical report commissioned by the European Centre for Disease Prevention and Control (ECDC) details the public health benefits and context for partner notification within Europe [5]. The report notes that, some countries have wide-ranging legal obligations to enforce partner notification, others have laws that are not enforced; and there are countries in the region with no such laws at all. There are laws or regulations in eleven European countries that make partner notification compulsory for the healthcare provider, the patient or both [5].

Second, the CDC recommendations for partner services for Human Immunodeficiency Virus (HIV) infection and three other STIs (syphilis, gonorrhea, and chlamydial infection) provide implementation guidance in the United States [6]. As used in the recommendations, “partner services” is broad, with partner notification as a critical component. Functions of partner services include prevention counseling, HIV and STD testing, treatment or linkage to medical care, linkage or referral to other services.

Third, the WHO in April 2012 issued guidance on couples HIV testing and counseling including antiretroviral therapy (ART) for treatment and prevention in sero-discordant couples [11]. Partner notification is a prominent feature of the guidelines.

Beyond this background, we present a review of the principles and goals of partner notification, partner notification methods and approaches, and the data behind partner notification.

### Review

We constructed searches with relevant key words in *OvidMedline,* and *Embase.* These were supplemented with ‘unrestricted searches” in Google Scholar. Citations of retrieved resources were also perused for additional resources. A careful study of these and other resources provided the background data for our analysis. The first search in OvidMedline with key words [HIV infections, partner notification or contact tracing, privacy] spanned the period 1946 to week 4 of September 2013 and yield 83 records. These were manually reviewed and reduced to 27. A related search in the same database replaced partner notification or contact tracing, with partner service or expedited partner therapy and yielded 27 records that were manually reviewed and reduced to six. A third search in Embase using same key words yield two resources. Both were relevant.

### Principles and goals of partner notification

Cognizant of the hypothesis that secrecy nurtures disease by providing an environment conducive to the spread of infection, Gostin and Hodge [14] note that one of the earliest recorded public health strategies for STI prevention was to pierce the veil of secrecy surrounding these hidden diseases by notifying sexual partners of infected patients. This was supported by the moral theory that sexual partners could take precautions and seek medical treatment if the risk of infection was disclosed. Once the risks of infection were identified, the incidence of STD infection would decline suggestively as infected persons reduced behaviors that placed them at risk for disease [12]. The following principles as set out in all partner notification guidelines, serve as the foundation for providing partner notification services: Such services ought to be client-centered, confidential, voluntary, free, evidence-based, comprehensive and integrative, culturally, and developmentally appropriate, accessible and available to all [5,6].

Every partner notification program seeks to maximize access to HIV prevention services by providing all infected persons (index patients) with support to ensure that the partners are confidentially informed of their exposure, tested, and linked to medical care. At the community level, partner notification seeks to reduce future rates of transmission in line with the current treatment as prevention arguments [15-17]. By linking partners to care and reducing their viral load, the community viral load/level of infectiousness is reduced [18-22]. Also, by identifying persons with previously undiagnosed HIV infection through partner notification and linking them to medical care services, and possibly to ART, partner notification services help reduce transmission within the community.

### Partner notification methods and approaches

Conventionally, four strategies have been used in implementing partner notification: provider referral, self-referral, contract referral, and dual referral.

Provider referral notification involves a partner being notified of their possible exposure by a health professional who has been specifically trained to locate and notify partners. The healthcare worker then links the partners to medical, prevention, and support services while protecting the confidentiality of the index patient.

In a patient referral, the index patient takes responsibility for informing their sexual partner(s) of their possible exposure to an STI and for referring them to services. Introduced in the 1970s in response to high levels of gonococcal infection [23], patient referral has since been widely used for a wide range of STIs [5,6,24].

Contract referral notification involves index patients selecting specific partners they prefer to notify and agreeing to a specific time frame in which they will do so. Patients agree that if they do not notify the selected partners within the established time frame, the provider will notify the partners. Dual referral notification involves an index patient and the provider (or third party) jointly notifying a partner of exposure [5]. The data in support of partner notification are summarized below.

### The data behind partner notification’ in the context of HIV

Credible data exist in support of partner notification’s role in HIV prevention-care cascade. We present some of these data. In the US for example, when syphilis prevalence peaked in 1990, partner notification among other national public health interventions contributed to its reduction and, later, elimination [25]. The efforts were adjudged to be cost efficient [25,26]. Also, evaluation of 10 years of data from the New York program for notification and referral services for gonorrhea, as well as of other program data showed a reduction in gonorrhea prevalence [27,28].

Other calls for the implementation of partner notification exist. For instance, it is argued that, of the approximately 1–1.2 million persons living with HIV infection in the United States, 25% are not aware of their infection; transmission from persons not aware of their infection accounts for 54%-70% of new infections [29,30]. A case is therefore made for partner notification given that it effectively identifies persons with previously undiagnosed HIV infection [31].

We present now the interests and counter interests in partner notification.

### The interests in partner notification

Partner notification as a public health intervention, is implemented by governments to, among other reasons, reduce the incidence of new cases of STIs in individuals and in the community [5]. However, such interests of the government sometimes conflict with those of the individual. Two cases in the United States, the Jew Ho v. Williamson (1900) and Jacobson v. Massachusetts (1905) speak to such conflicting interests in public health practice. In his distillation of Jacobson v. Massachusetts, Gostin notes that governmental interests, though grounded firmly in statutory directives such as the police power^a^, are not absolute [32]. Gostin clarifies that police powers cannot be exercised in a manner that violates the constitutional rights of individuals.

Resolution of these conflicted interests may call for legal jurisprudence, intervention by ethicists/bioethicists, or both. “Interest Analysis” has been applied in the past to resolve related conflicting interests [33,34]. Coined by Brainerd Currie in 1963, and popularized by Gostin and Hodge [14], interest analysis, as we will apply in this paper will entail a disinterested analysis of the various interests in partner notification, and not in legal jurisprudence. The interests at play here are the interests of the index patient, the interests of partner, and the interest of the public health profession/al. These interests are discussed.

### Public health interests in partner notification

The public health interests of partner notification are justified in a number of ways. First, such interests are premised on the theory that partners are entitled to knowledge. This theory posits that “knowledge empowers individuals to avoid continuing risks; knowledge of infection allows for early treatment; and that knowledgeable partners can adapt their behavior to prevent further transmission of infection to others” [14,35]. Available evidence shows that people are less likely to have unprotected sex once they are aware of their HIV status [36,37]. Additionally, equipping public health officials with knowledge helps to prevent infection of and/or to treat sexual partners – control the spread of STI and its attendant consequences in the general public [35,36]. A review by Christie and Kendall show that prevalence of HIV among identified contacts ranges from 15% to 30%, with the majority of contacts being unaware of their possible exposure. Their conclusions concur with afore cited works [25-28] that, that partner notification is an effective public health intervention [38]. Thus partner notification programs offer substantial benefits to three principal groups: persons infected with HIV, their partners, and the community [6]. Presented this way, public health interest may be viewed as coinciding with those of the index patient, the interests of his/her partner, and the interests of the community as a whole.

### Individual level interests

While governmental interests in partner notification may be equated to the interest of all, persons infected with HIV have individualized or counter interests [14]. For instance, an infected person may desire to maintain the privacy of their health status from unnecessary disclosure because of the negative impacts of disclosure (done consensually or otherwise). Emotional or physical abuse by or against the index patient, domestic violence of a physical or psychological nature as well as dissolution of a long-standing relationship [39,40] have been associated with partner notification. Theresa Yuricic [41] writes about how stigma particularly within the African-American community potentially decreases the efficacy of partner notification laws in this population. Disclosure of HIV status she notes can result in social stigma among their family and friends. Yuricic further discusses how disclosure makes them vulnerable to discrimination in employment, housing, and insurance [41]. Angela Nicoletti [42] enumerates other interests including fear of losing family support. Negative impacts of partner notification on infant feeding have been recording in Ghana [43]. It may thus seem reasonable, given these potential and real negative implications on the index patient, to respect their interests when systems or health personnel are unable to assure their security. In this regard, screening for likelihood of violence and/or other serious negative consequences on the index patient ought to be an important part of the partner notification process.

### The bioethicist’s interests in partner notification

The interest of the bioethicist in this matter is to facilitate a moral resolution of the conflicted interests. S/he is interested in providing nuanced answers to such questions as whether or not index patients or healthcare providers have a moral duty to inform partners of their exposure risk, whether or not the healthcare provider’s relationship with the index patient takes priority over the obligation to protect others from the patient’s infection, or just how important is the partner’s right to know that he or she may be at risk? These are not ordinary questions. Gostin and Hodge [14] note that while the law provides its own answers to these questions, ethical reasoning or moral resolution is important. The fundamental conceptual problem is the difficulty in telling which of the claims (the index patient’s or the partner’s) should take precedence. Our analysis draws on Kass and Gielen’s [14,44] as well as Gostin and Hodge’s [14] earlier works. Like Kass and Gielen, we believe that principles-based analysis may help in the bioethicist’s search for answers to the above questions.

#### Partner notification and the principle of beneficence

To Tom L. Beauchamp, and James Franklin Childress, beneficence means that persons have the responsibility to do good for others, to prevent harm to others, or, at the very least, to avoid directly harming others [45]. In the context of partner notification, promoting beneficence would mean carefully balancing the harms and benefits of partner notification – balancing the various interests. Without a doubt, any effort that seeks to prevent new HIV infections is pregnant with beneficence. An important question that earlier commentators have asked is: “how ethically effective partner notification programs are in achieving this goal” [44]. To provide a reasonable response as to the extent partner notification programs satisfy the principle of beneficence, Kass and Gielen [44] suggest a set of five assumptions all of which need to be met. These are:

1. The index patients must know and be willing to disclose the names of their contacts, and it must be possible to locate the contacts;
2. a significant proportion of the persons contacted must not have known already that they had been exposed to HIV, and they must have been practicing unsafe practices before and be willing to change to safer practices now;
3. A significant proportion of the persons contacted must be willing to be tested;
4. Some number of those found to be HIV-infected will not have known already that they were infected;
5. Some number of persons found to be HIV-infected must have been practicing unsafe practices before being contacted who will change to safer practices after being informed of their potential exposure.

We suggest a sixth assumption – partners who willingly accept to be tested when found to be HIV+ would have unfettered access to HIV treatment and prevention services for self and other partners. We note however, that arguments in support of this newly introduced assumption, needs to be nuanced by discussions on the ethical tensions associated with HIV treatment commodity allocation dynamics. For instance in settings where ARVs are very scarce, arguments concerning whether or not it is ethical to divert scarce ARVs from PLHIV who meet both the implicit adherence and the explicit medical eligibility criteria to others who do not. In support of the former, Elliot Marseille and colleagues argue forcefully for the supremacy of prevention over treatment even when it means denying treatment to medically eligible PLHIV [46]. Brock and Wikler [47] also argue that ‘the strongest moral imperative directs us to give priority to saving the most lives even if this means lowering the priority given to the goal of universal access to treatment, to provide maximum protection from HIV infection’. Macklin and Cowan on the contrary argue that it will be unethical to ‘deliberately watch patients with treatable AIDS worsen and die, if medications for treatment are diverted to for this purpose [48].

The extent to which Kass and Gielen’s assumptions were met then or now is unknown. There are no published studies evaluating partner notification programs on the terms stated above. Studies whose findings could qualify as near approximations suggest that partners who were notified were significantly more likely to modify their sexual behavior and to avoid intercourse than those who had not been notified [49,50]. Other studies show that partners believe their chances for preserving their individual health rely in substantial part on notification of risk [17,37]. It follows, they argue, that universal notification is a plus to preserving and promoting public health. Yet, many infected persons, particularly women, counter these observations by documenting the costs of partner notification in the form of domestic violence and abuse [37,39,40].

Determining the balance between these competing interests is not easy. In its current application, partner notification may not represent a proper balance – “beneficence-wise”. This paper’s stance based on a conditional consequentialist reasoning supports the roll out of partner notification. In line with current arguments for treatment as prevention (TasP) and pre-exposure prophylaxis (PrEP), partner notification should have an additional condition of ensuring that index patients and their notified partners have access to HIV treatment and prevention commodities. The second condition (which entails prior screening for possible partner abuse) is inked in the latest guidelines by the CDC [6].

#### Partner notification and the principle of justice

Justice, according to Beauchamp and Childress, requires that people be treated fairly [45]. That is restrictions cannot be imposed on, or benefits provided to, one person or one group of people when another similarly-situated person or group is treated differently without adequate justification [45]. Any partner notification program that mirrors the above is morally indefensible. For instance, programs that prioritize men who have sex with men (MSM), female sex workers, or index patients suspected to have concurrent multiple sex partners or selectively notifies their partners should have a sound scientific justification. Such sound justifications would include for example, evidence showing that the group is significantly at greater risk of transmitting or acquiring the infection – in the case of MSM and sex workers. In that case disproportionate benefits/risks will not be unjust.

Per evidence [6,11], partner notification policies may be said to be just, but not necessarily practicable or fair implementation-wise. Pottker-Fishel’s analysis of legal cases, state statutes and other literature shows that many states’ statutes on partner notification are ineffective and burdensome, placing too much responsibility on health care providers [51]. There are also other studies that suggest that physicians may execute their responsibilities with regard to partner notification inconsistently depending on the demographics of their HIV-infected patients. For instance, findings of a national survey of US physicians that assessed among others partner management, and clinical practices for STIs including HIV show that variables such as gender, race, and sexual orientation would influence a decision to maintain or to violate patient confidentiality [52].

From the female index patient’s perspective, partner notification programs while beneficial for women who are HIV-negative, may be harmful for HIV-positive women (costs such as domestic violence and other abuses have been discussed in this paper). Some commentators have argued that since uninfected women stand to benefit more from the implementation of partner notification programs than infected women, partner notification may be unjust. Second, given that women are at greater risk of heterosexual transmission than are men, it may be argued that heterosexual women are more likely than heterosexual men to benefit from partner notification. These are relevant justice questions. We nevertheless, believe that the disproportionate benefits in these instances are not unjust, as they are also disproportionate risks.

#### Partner notification and the principle of respect for autonomy

The third bioethical principle of respect for autonomy, means that people must be respected as autonomous agents who have the right to make decisions for themselves without interference from others [45]. Some commentators have argued that, this principle creates a moral right to decide whether, when, and to whom one should release personal information. On the one hand, if this principle is followed to the letter, and in the context of partner notification, an index patient in his/her quest to preserve his/her privacy can simply choose not to disclose his/her partners’ names. On the other hand, a partner’s normative claim about invasion of autonomy is also reasonable. Partners, according to Gostin and Hodge [14] can appeal to autonomy in claiming a right to know. Partners could argue that they cannot make rational, or autonomous choices in the absence of relevant information. Indeed, Sarah Conly’s recent work titled *“Against Autonomy: Justifying Coercive Paternalism”* [53] would seem to favor the partner’s claim. The autonomous index patient’s right to engage in behaviors of his/her choosing does not extend to behaviors that can result in serious harm to partner(s). On the basis of these, an index patient may have no legitimate moral claim to maintain his/her confidentiality when doing so has a real potential to harm (HIV infection and its associated consequences in this case).

## Conclusions

This review notes that, partner notification is grounded in credible evidence, and is sound in public health standings, but has multivalent ethical tensions and interests. Our analysis shows that while governmental interests are based on evidence and in some instances backed by state statutes, they are not absolute in comparison with the interests of the index patient. By all means, the autonomy of the index patient should be respected and so should the interest of his/her partners, and the community’s at large. Against this backdrop, we reiterate that any effort to morally balance the benefits of partner notification with its burdens ought to first recognize the multivalent nature of the interests at play.

## Endnote

^a^The power of the state to make laws to secure the comfort, convenience, peace, and health of the community.

## Abbreviations

ART: Antiretroviral therapy
CDC: United States Centers for Disease Control and Prevention
ECDC: European Centre for Disease Prevention and Control
HIV: Human Immunodeficiency Virus
PEP: Post-exposure prophylaxis
PrEP: Pre-exposure prophylaxis
STIs: Sexually transmissible infections
TasP: Treatment as prevention
UNAIDS: The Joint United Nations Programme on HIV/AIDS
WHO: World Health Organization.
